# Efficacy of a Transdiagnostic internet-based treatment for emotional disorders with a specific component to address positive affect: Study protocol for a randomized controlled trial

**DOI:** 10.1186/s12888-017-1297-z

**Published:** 2017-04-20

**Authors:** Amanda Díaz-García, Alberto González-Robles, Javier Fernández-Álvarez, Azucena García-Palacios, Rosa María Baños, Cristina Botella

**Affiliations:** 1 Universitat Jaume I, Castellón, Spain; 2 Universidad de Valencia, Valencia, Spain; 3 CIBER Fisiopatología Obesidad y Nutrición (CIBERObn), Instituto Salud Carlos III, Madrid, Spain

**Keywords:** Transdiagnostic, Internet, Positive affectivity, Emotional disorders, Depression, Anxiety, Emotion regulation, Randomized controlled trial

## Abstract

**Background:**

Emotional disorders (ED) are among the most prevalent mental disorders. However, less than 50% of people suffering from ED receive the appropriate treatment. This situation has led to the development of new intervention proposals based on the transdiagnostic perspective, which tries to address the underlying processes common to ED. Most of these programs focus primarily on down-regulating negative affectivity, rather than increasing strengths and up-regulating positive affectivity. The data suggest the existence of disturbances in positive affectivity in these disorders, and so new interventions focusing on these problems are greatly needed. It is also essential to provide assistance to all the people in need. Information and Communication Technologies can be very useful. This study aims to evaluate the efficacy of a transdiagnostic Internet-based treatment for ED in a community sample. The protocol includes traditional CBT components, as well as a specific component to address positive affect. We intend to test this protocol, including this specific component or not, versus a waiting list control group. Moreover, we aim to test the differential effect of this specific component, and study the effectiveness (in terms of patients’ acceptance) of using a self-applied Internet-based program. This paper presents the study protocol.

**Methods:**

The study is a randomized controlled trial. 207 participants will be randomly assigned to: a)*Transdiagnostic Internet-based protocol (TIBP),* b)*Transdiagnostic Internet-based protocol + positive affect component (TIBP + PA),* or c)a Waiting List control group (WL). Primary outcomes measures will be the BDI-II, the BAI, and the PANAS. Secondary outcomes will include diagnosis-specific measures of the principal disorder. Participants’ treatment acceptance will also be measured. Participants will be assessed at pre-, post-treatment, and 3- and 12- month follow-ups. The data will be analyzed based on the Intention-to-treat principle. Per protocol analyses will also be performed.

**Discussion:**

To the best of our knowledge, this is the first study of a transdiagnostic Internet-based treatment for ED with a specific component to up-regulate positive affectivity. This intervention could contribute to improve the efficiency and effectiveness of current treatment programs for ED, promote the dissemination of EBTs, and help to decrease the high prevalence of ED.

**Trial registration:**

ClinicalTrial.gov: NCT02578758. Registered 15 October 2015.

## Background

### Introduction

Lifetime prevalence estimates for emotional disorders (ED), defined as anxiety and unipolar mood disorders, are quite high (anxiety disorders, 28.8%; mood disorders, 20.8%), with comorbidity rates ranging between 40 and 80% [1]. These disorders disrupt the lives of millions of people each year, and they are one of the main causes of disability worldwide [2, 3].

In the past few decades, evidence-based psychological treatments (EBTs) have been shown to be effective in the treatment of ED [4]. This development can be considered a significant advance in addressing the large worldwide treatment needs [5]. However, the scale of these treatments is not sufficient to reduce the disease burden of mental disorders [6]. Less than 50% of people suffering from ED receive adequate treatment [7], and this percentage is much lower in adolescents, older adults, people with a lower socio-economic status, and people from ethnic minorities [8].

These low levels of successful dissemination of EBTs can be explained by their costs, the duration of the treatments, and the lack of well-qualified professionals [9]. This is especially problematic in everyday clinical practice and can explain why EBTs are under-utilized [10]. It is not surprising, therefore, that this continued lack of widespread availability of EBTs has raised the need to implement innovative and even radical solutions to ensure that the aid reaches all those in need [8, 11, 12]. There is a compelling need for approaches that go beyond “one-to-one” psychotherapy (either with a patient, a family, or a group) and develop a new portfolio to administer EBTs [6].

Different EBTs targeting specific anxiety and mood disorders have been developed [13–16]. Each diagnosis-specific treatment manual requires the use of separate handbooks, workbook, and protocols, and therapists must be trained in the use of each of them, which may hinder widespread dissemination of EBTs [9].

In recent years, new proposals have emerged for transdiagnostic treatments that emphasize the essential processes underlying different disorders. In the field of ED, studies emphasize that these disorders share important characteristics, and that this overlap emerges from common biological and psychological vulnerabilities [14, 17, 18]. Barlow summarizes the commonalities in the etiology of ED in the model referred to as “triple vulnerabilities”. This theory encompasses a generalized biological vulnerability (involving nonspecific genetic contributions to the development of anxiety and negative affect), a generalized psychological vulnerability (associated with early life experiences under certain conditions), and a specific psychological vulnerability emerging from early learning [14]. There is evidence that the two generalized vulnerabilities are involved in the development and expression of the ED [18, 19].

People with ED have higher levels of neuroticism/negative affect/behavioral inhibition (N/NA/BI) [18], and they experience negative emotions more intensely and frequently [20, 21], accept emotional experiences to a lesser extent [22], associate the experience of living with more negative emotions [23], use cognitive and behavioral strategies to reduce the impact of negative emotions [24], and show intolerance to uncertainty, leading to an increase in negative affect [25]. In short, people with ED tend to react negatively to their emotions and are more likely to use maladaptive emotion regulation strategies. These strategies, in turn, increase the frequency/intensity of negative emotions. Some authors have argued that this functional relationship may be driven by neuroticism, which would be the core of the ED [26].

By contrast, the role of extraversion/positive affect/behavioral activation (E/PA/BA) in ED has also been pointed out. A meta-analysis indicated that most individuals with an ED show low levels of E/BA [27]. The data suggest that alterations in PA are observed in many disorders [28]. Low levels of PA predict the onset of depression [29], dampen positive emotions, are maladaptive, and increase the severity of the problem [30], whereas high PA is associated with better health, both physical and psychological, and greater well-being [31]. Despite the importance of disturbances in positive affectivity in ED, few studies focus on promoting PA.

Based on the transdiagnostic perspective, Barlow’s team designed the Unified Protocol (UP): a transdiagnostic, emotion-focused, cognitive-behavioral treatment for ED that emphasizes the role of emotion regulation [9, 26, 32]. The UP focuses on four essential aspects that have the general purpose of down-regulating NA: addressing emotional avoidance, promoting cognitive flexibility, and facilitating exposure to avoided situations and sensations. Moreover, it places special emphasis on increasing present-focused emotional awareness. The UP has been tested, and results indicate that it is effective [33], with improvements maintained at 18-month follow-up [34]. Moreover, the effect of the UP has been shown on the two temperament dimensions of N/BI and E/BA [35].

The data suggest that a transdiagnostic treatment for ED might be more widely effective across diverse mental health problems, in other words, treatments aimed at addressing different disorders with a single protocol [36]. Some meta-analyses have been conducted on the efficacy of transdiagnostic protocols for anxiety disorders [37] and for anxiety and/or depression [38–40].

Nevertheless, these transdiagnostic protocols have focused on reducing NA, but less attention has been paid to promoting PA or modifying risk factors. As the World Health Organization’s definition of mental health expresses, mental health is more than just the absence of mental illness [41]. It is not surprising, therefore, that well-being and positive functioning are considered core elements of mental health. As Southwick and Charney [42] point out, the use of procedures to promote resilience (such as positive emotions and optimism) can be useful in the treating ED and in generating protective factors. The benefits may be associated with a reduction in the risk of developing mental symptoms and disorders [43, 44]. However, as stated above, interventions that include components to up-regulate PA have been missing or very scarce in the clinical setting.

Taking all this into consideration, it is necessary to develop and test treatment components focused on enhancing protective factors and resilience and mitigating risk factors. Literature has highlighted the potential importance of positive emotionality as a treatment component [45, 46].

To date, the dominant delivery format in psychotherapy has been individual face-to-face contact; however, it is much more expensive and time-consuming than other formats, such as guided self-help and Internet-based treatments [47]. Recently, research has shown that Information and Communication Technologies (ICT) can facilitate the availability of EBTs [6, 48, 49]. Specifically, some literature suggests that the Internet can be used for the assessment and treatment of clinical conditions [50]. Internet-based treatments are interventions conducted over the Internet with more or less therapist involvement and support [51]. The evidence strongly suggests that Internet-based treatments are effective in the treatment of depression and anxiety disorders [52]. Moreover, data from meta-analyses reveal that these interventions are as efficacious as face-to-face traditional treatments [53–55].

We have developed an online psychological treatment protocol for individuals with a diagnosis of ED [56], major depression disorder (MDD), dysthymic disorder (DD), obsessive-compulsive disorder (OCD), and four anxiety disorders: panic disorder (PD), agoraphobia (AG), generalized anxiety disorder (GAD), social anxiety disorder (SAD), anxiety disorder not otherwise specified (ADNOS), and (unipolar) mood disorder not otherwise specified (MDNOS).

This treatment protocol includes two types of components: one based on classical perspectives for down-regulating NA and the other aimed at up-regulating PA. The protocol can be applied either in its traditional format (*Transdiagnostic Internet-based Protocol –TIBP–)* or by including both of these components (*Transdiagnostic Internet-based Protocol + Positive Affect Component –TIBP + PA–*). Moreover, we have developed an adaptation of the treatment protocol that can be applied online over the Internet. We can thus reach community samples, that is, people who suffer from an ED but are not receiving primary or specialized care.

We intend to study the effect of both treatments in terms of efficacy for depressive and anxious symptomatology. Moreover, we aim to assess the effects of the specific treatment component designed for up-regulating PA. Finally, the effectiveness of the Internet-based program developed to apply the treatment protocol over the Internet with minimal support by the clinician will be studied. We hypothesize that: a) both self-applied protocol modalities (*TIBP* and *TIBP + PA*) will be more effective than the waiting list control condition in the treatment of ED; b) both interventions will result in significant improvements in depressive and anxious symptomatology at post- treatment, and these results will be maintained at 3- and 12- month follow-ups; c) the *TIBP + PA* will significantly outperform the *TIBP* group on PA measures; and d) both protocols will be well accepted, with no statistical differences between conditions. In this article, we present the study protocol.

## Methods/Design

### Study design

**Fig. 1:**
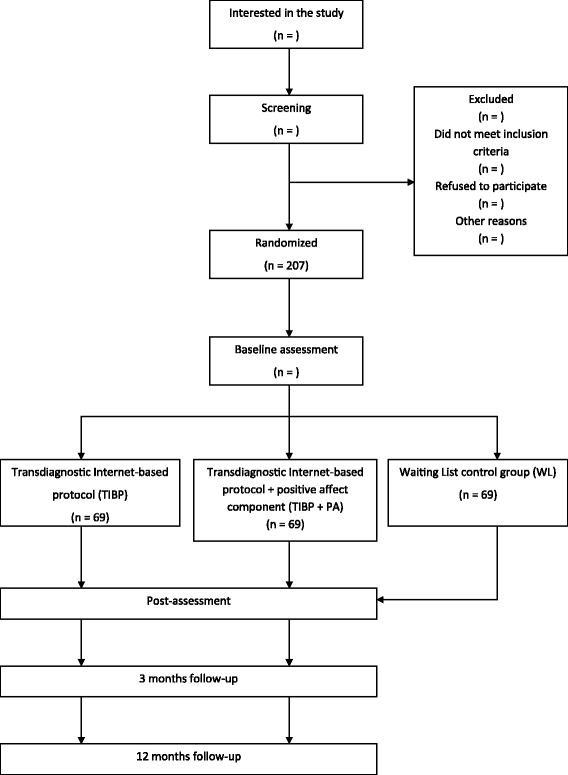
Flowchart of participants

A three-armed randomized controlled trial (RCT) will be conducted. Participants will be randomly allocated to one of three conditions: a) *Transdiagnostic Internet-based protocol (TIBP)*, b) *Transdiagnostic Internet-based Protocol + Positive Affect component (TIBP + PA),* and c) *Waiting List control condition (WL)*. For ethical reasons, participants in the control condition will be offered the possibility of receiving the treatment protocol. Block randomization will be performed within each stratum in order to ensure that all primary diagnoses are equally represented across conditions. Measures will be taken at post-randomization, after the treatment, and at 3- and 12-month follow-ups, in order to test whether the improvements achieved during the therapy are maintained in the long term.The study flowchart is shown in Fig. 1. The study will be conducted following the CONSORT statement (Consolidated Standards of Reporting Trials, http://www.consort-statement.org) [57, 58] the CONSORT-EHEALTH guidelines [59], and the SPIRIT guidelines (Standard Protocol Items: Recommendations for Interventional Trials) [60, 61].

### Sample size

To determine the sample size, the effect sizes found in the literature have been considered. An RCT using the UP in a traditional face-to-face approach [33] obtained effect sizes of 0.56 for anxiety, measured with the BAI, and 1.11 for depression, measured with BDI-II. The mean effect size indicated in a recent meta-analysis [39] comparing transdiagnostic computerized cognitive-behavior therapy (CBT) to waitlist control was g = .93 for anxiety and g = .88 for depression.

Adopting a more conservative approach than those of the aforementioned studies, we assumed an effect size of 0.5 (Hedges’ g), which, following Cohen [62], can be considered an effect size of medium magnitude. As our design included three experimental conditions (*TIBP*, *TIBP + PA*, *WL*), a between-groups one-way ANOVA was assumed for the statistical analyses. Therefore, calculations of the necessary sample size cannot be based on Hedges’ *g* index for effect size, but rather on the *f* index [62]. Following Cohen [62], *f* = 0.25 represents an effect size of medium magnitude (and equivalent to *g* = 0.5). Thus, considering an alpha of .05 and a statistical power of .80, the total sample size required to warrant these conditions contains 159 participants (53 participants per group). To control the maximum possible loss of subjects during treatment, based on the literature about Internet-based treatments, a 30% dropout rate is expected [53, 63]. Thus, the required sample size should have 207 participants in all (69 participants per group). These calculations were accomplished with the software program G*Power 3.1 [64].

### Study population, recruitment and eligibility criteria

The clinical trial will be conducted in a community sample of patients diagnosed with the aforementioned disorders. Participants will be adult volunteers who contact us because they are interested in the study (personal visits or phone calls to the emotional disorders university clinic, emails, or leaving their data on a website specially prepared for this purpose). Potential participants will be attended to by a psychologist who will describe the study characteristics to them. All the psychologists working on this study will have at least a master’s degree in Clinical Psychology, with experience in the diagnosis, psychological assessment, and application of EBTs for ED.

The psychologist will clarify any doubts, ensure that the participant has read the information about the study, and make sure that s/he has understood the three experimental conditions. People interested in participating will sign an online informed consent and be assessed taking into account all the inclusion criteria. Inclusion and exclusion criteria are shown in Table 1. If the patient fulfills all the study criteria, the researcher will contact an independent researcher to implement randomization. This researcher will be unaware of the characteristics of the study. Randomization will be performed using weighted random allocation [65] in order to take into account the clinical features (different diagnoses) and, thus, obtain a homogeneous distribution in the three experimental conditions. Participants agree to participate before finding out to which treatment they will be allocated. All participants will be free to withdraw from the treatment at any time.

**Table 1:** Inclusion and exclusion criteria

Inclusion criteria	Exclusion criteria
Minimal age of 18 years	Suffering from Schizophrenia, bipolar disorder, or alcohol and/or substance dependence disorder
Meeting the DSM-IV diagnostic criteria for ED	High risk of suicide
Ability to understand and read Spanish	Medical disease/condition that prevents the participant from carrying out the psychological treatment
Access to Internet at home and having an email address	Receiving another psychological treatment during the study
Providing online informed consent	An increase and/or change in the pharmacological treatment (in the case of receiving) during the study period

### Ethics

The study follows the guidelines of the Declaration Helsinki and existing guidelines in Spain and the European Union for the protection of patients in clinical trials. As noted, all participants will be volunteers, and they will sign the online informed consent to form part of the study once it has been explained to them. Participants will have the possibility of withdrawing from the study at any time. The recruitment of participants will be carried out by qualified personnel from a clinical point of view. Participants allocated to the *WL* condition will be offered the opportunity to receive the *TIBP* or the *TIBP + PA* intervention after the waiting time has ended. Based on the literature, no special difficulties are expected. However, any undesired event would not only mean the participant’s departure from the trial, but s/he would also be offered the possibility of receiving psychological care at the Emotional Disorder Clinic in Universitat Jaume I, or of being referred if his/her medical condition required it.

A fundamental aspect in a project of this nature is data protection. To protect information, strategies using personal passwords and data via AES encryption (AES-256; Advanced Encryption Standard) will be used. Personal data will be replaced by codes and data, which must be collected by clinicians (e.g. age, sex, address, and phone), stored separately from other data, and only made available to researchers responsible for the study, always protecting the right to privacy.

The study has been approved by the Ethics Committee of Universitat Jaume I (Castellón, Spain). The trial was registered at clinicalstrial.gov as NCT02578758.

### Interventions

We have developed a manualized protocol, based on the transdiagnostic perspective for the treatment of ED: MDD, DD, OCD, PD, AG, GAD, SAD, ADNOS and MDNOS. The treatment protocol is structured in a patient handbook and a therapist handbook (Botella C, García-Palacios A, Quero S, Baños R. A Transdiagnostic Treatment for Emotion Disorders: Manualized Treatment Protocol, unpublished). The modules in each intervention protocol are described briefly in the following section.

We have adapted this protocol from classic perspectives derived from the UP [9, 17] and some strategies from Marsha Linehan’s protocol [66]. The protocol includes the following core components mainly addressed to down-regulate NA: present-focused emotional awareness and acceptance, cognitive flexibility, behavioral and emotional avoidance patterns, and interoceptive and situational exposure. The protocol also includes traditional therapeutic components of evidence-based treatment for ED (Psychoeducation, Motivation for change, and Relapse prevention). In addition, in order to promote psychological strengths and enhance well-being [67], we have included a PA-regulation component based on behavioral activation strategies [68], strategies to promote pleasant and significant activities linked to values and life goals, and strategies to enhance personal strengths, positive feelings, positive cognitions, and positive behavior [67, 69]. This component also includes the Well-being Therapy (WBT) [70, 71] strategy of identifying examples of well-being and negative cognitions (“interrupting thoughts”) that interfere with these moments of well-being, in order to modify these interfering cognitions. Some notions of Fredickson’s Broaden-and-Build Theory are also included to explain the mechanisms behind positive emotions [31].

#### Transdiagnostic Internet-based protocol (TIBP)

This intervention protocol consists of twelve more traditional modules mainly designed to down-regulate NA. Each module has specific objectives:

- *M1. Emotional disorders and emotion regulation.* The purpose of this module is to provide information about the central role of emotion regulation in emotional disorders. Brief descriptions of the program modules, as well as videos with examples of people suffering from different ED, are also presented.
- *M2. Motivation for change.* The objective of this module is to enhance motivation for change by recognizing that attitudes towards change can be ambivalent and that motivation fluctuates. The main objective is to analyze the advantages and disadvantages of changing, emphasize the importance of being motivated, and highlight the importance of establishing significant life goals.
- *M3. Understanding the role of emotions.* This module provides information about the adaptive roles and functions of emotions. It also shows the three-component model of emotions.
- *M4. The acceptance of emotional experiences.* This module focuses on the awareness and role of acceptance of emotional experiences, as well as their importance in the treatment.
- *M5. Practicing acceptance.* The objective of this module is to continue to learn about the acceptance of emotional experiences and increase awareness of physical sensations, thoughts, emotions, and daily activities.
- *M6. Learning to be flexible.* This module teaches participants to be more cognitively flexible in order to see life situations from different perspectives, showing the importance of maladaptive ways of thinking and learning how to identify them in the maintenance of emotional disorders.
- *M7. Practicing cognitive flexibility.* This module aims to teach the patients how to modify maladaptive ways of thinking. It also provides information about intrusive thoughts and how to deal with them.
- *M8. Emotional avoidance.* The aim is to teach the patients emotion avoidance strategies that contribute to the maintenance of emotional disorders.
- *M9. Emotion Driven Behaviors (EDBs).* The aim is for patients to learn the concept of EDBs and replace their own maladaptive EDBs with other more adaptive behaviors.
- *M10. Accepting and facing physical sensations.* The objectives are to teach the patients the role of physical sensations in their emotional response and train them in interoceptive exposure, in order to increase tolerance and promote habituation to physical sensations.
- *M11. Facing emotions in the contexts where they occur.* This module aims to increase tolerance to emotions and reduce avoidance behavior. The purpose is to construct exposure hierarchies to help patients to begin to face the avoided situations that contribute to the maintenance of the problem.

#### Transdiagnostic Internet-based protocol + positive affect component (TIBP + PA)

This intervention protocol is made up of 16 therapeutic modules. The first 12 modules have been described above in the first condition *- (TIBP) -* and the other 4 are aimed to up-regulate PA by understanding and capturing positive emotions, and training adaptive positive emotion regulation strategies. These four modules are described as follows:

- *M12. Learning to move on.* This module focuses on the role of behavioral activation. The aim is to teach the importance of ‘moving on’ in acquiring a proper level of activity and involvement in life. It motivates the patient to get involved in meaningful activities and become engaged in his/her life.
- *M13. Learning to enjoy.* This module helps the patient to see the importance of positive emotions and teaches procedures that generate positive experiences, promoting involvement in pleasant and significant activities and contact with others. This module involves enjoying positive experiences and “savoring” positive aspects of life in order to enhance wellbeing.
- *M14. Learning to live.* This module takes a further step in enhancing PA, understanding the importance of identifying the individual’s own psychological strengths, and selecting and carrying out meaningful activities linked to values and goals in life. This module provides strategies to achieve psychological well-being and resilience. The aim is to improve the individual’ abilities and live a life full of purpose and meaning.
- *M15. Living and learning.* This module focuses on developing an action plan to boost the individual’s psychological strengths. It focuses on the importance of developing and enhancing one’s own strengths and starting to work for life and the future.
- *M16. Relapse prevention (this module is the same for both conditions: (TIBP) and (TIBP + PA).* It aims to strengthen the strategies learned throughout the program, schedule future practice, and teach how to identify and cope with future high- risk situations.

### Adaptation to the web

This protocol has been adapted to a multimedia web platform (video, images, etc.) and can be applied over the Internet (https://www.psicologiaytecnologia.com/). It is developed for optimal use on a PC or a tablet, and it allows individuals to do the modules from their home and at their own pace. The ease of use of the program has been strengthened because it presents a streamlined navigation, which allows users with less experience in handling new technologies to know where they are and how to keep moving forward at any time. The web adaptation of the protocol is the same in both conditions (TIBP and TIBP + PA), with the only difference being the inclusion or not of the modules that contain the PA-regulation component.

Duration of the program can vary among users, and participants in both treatment conditions have access to the protocol for a maximum period of 18 weeks. The program recommends working on one module for at least one entire week, and it sends messages encouraging the patient to continue to work to benefit from it. In both conditions, the modules include exercises and tasks to practice each technique and skill. Moreover, the platform is ready to welcome all participants with information about the treatment and its objectives, as well as general information and recommendations about how to benefit from it.

Regarding the assessment of patients within the program, after the “Welcome”, initial online questionnaires are presented as the pre-treatment assessment. In addition, post-module brief questionnaires to evaluate anxiety, depression, and positive/negative affect (OASIS, ODSIS, PANAS) are also filled out throughout the program. A noteworthy aspect of the program is that it allows the therapist to have access to all this information and receive an alert if the patient’s condition worsens. The program includes suicide risk alarms that consist of an email to the clinical team with information about a high risk of suicide. These alarms are generated when the system detects that the participant rated high on the suicidal ideation items. It allows the clinician to contact the patient and evaluate the actions that should be taken to protect him/her. Finally, after the treatment, the participants complete the online assessment in the post- and follow-up periods.

All the modules are sequential, which allows the participant to move through the program step by step. The web platform was designed to optimize the understanding of the modules’ content. It has different multimedia elements (vignettes, videos, audios,...) that help the user to assimilate the different psychological techniques in the easiest way. The modules always follow the same structure: they start with questions related to the previous module, continue with the specific contents of the module, then propose the exercises, and finally present a series of simple self-assessment questions designed to verify whether the content presented has been understood correctly. In addition, at the end of each module, homework tasks are indicated to work on what was presented in the module. Performing these tasks helps to consolidate everything learned in the program. Participants have the opportunity to obtain printable documents (PDF) with summaries of each module.

The web platform has four complementary tools that appear on the main menu of the protocol: 1) “Home”, which is the starting point from which the participant can access the other sections of the protocol; 2) “Calendar”, which is a tool that allows the participant to know about pending or achieved tasks as well as his/her current point in the program; 3) “Review”, which allows the participant to review the treatment modules already completed; and 4) “How am I?”, which allows participants to monitor their progress through a set of graphs. It is a tool that provides feedback to participants about their evolution during the program with regard to their emotional distress (anxiety and sadness) and their positive and negative affectivity.

### Support

In each of the two treatment conditions, we will provide human support and ICT support to all participants.

Human support will be provided by weekly phone calls (maximum of 5 min) during the treatment period in order to resolve any difficulties or doubts about the use of the online protocol, or to remind them of the importance of doing the homework tasks and reviewing the treatment contents. During these weekly phone calls, we will also encourage participants to continue to use the protocol, and reinforce them for engaging in the treatment. At the end of the treatment period, participants will be reminded that they will be contacted again for follow-up assessments.

ICT support will consist of two weekly mobile phone text messages reminding participants of the importance of reviewing the modules and encouraging them to do the homework tasks. Some examples of these text messages are: “Hi there! Don’t hesitate to review the modules you’ve already completed if necessary. Remember, practice makes perfect!”or “Hi there! Don’t give up on your module tasks! Dedicate some time and effort to them. Remember, it’s ideal to complete one module each week”. These messages will be randomized, so that participants do not always receive the same content. A professional platform will be used to send these messages (www.trendoo.es). The online treatment also contains several multiple-choice questions about the contents seen in each module. The program immediately provides the participant with the correct feedback and a simple explanation. This aspect is repeated throughout all the treatment modules. In addition, the ICT support includes automatic e-mails with reminders to access the program when participants have not entered in the past 15 days.

### Measures

#### Diagnostic interview

*Mini International Neuropsychiatric Interview Version 5.0.0* (MINI) [72]. It is a short, structured, diagnostic psychiatric interview for DSM-IV and ICD-10 diagnoses. The MINI can be used by clinicians after a brief training session. Nonclinical interviewers should receive more intensive training. This interview has excellent inter-rater reliability (k = .88–1.00) and adequate concurrent validity with the Composite International Diagnostic Interview [73]. The MINI has been translated and validated in Spanish [74].

#### Primary outcomes measures

##### *Beck Depression Inventory (BDI-II)* [75]

It is one of the most widely-used questionnaires in the evaluation of depression severity. The BDI consists of 21 items, scored on a scale from 0 to 3, covering all the different symptoms of major depression disorder. The total score on this questionnaire can yield a maximum of 63 points. The instrument has shown good internal consistency (α = 0.76 to 0.95). The Spanish version of this instrument has also shown high internal consistency (α = 0.87) for both general and clinical populations (α = .89) [76].

##### *Beck Anxiety Inventory (BAI)* [77]

The BAI is a 21-item symptom checklist designed to assess anxiety symptoms. Each item is scored on a 0 to 3 point severity scale, and the total scores range between 0 and 63. The internal consistency of the BAI has been found to range from .85 to .94. The Spanish version of the BAI has shown high internal consistency (α = .93) [78].

##### *Positive and Negative Affect Scale (PANAS)* [79]

The PANAS consists of 20 items with a range from 1 (very slightly or not at all) to 5 (extremely). Respondents have to indicate the extent to which they experienced the feeling or emotion during the past few weeks. This scale evaluates two independent dimensions: PA and NA. The maximum score for each subscale is 50. The scale showed excellent internal consistency (α between .84 and .90) and convergent and discriminant validity. The Spanish version has demonstrated high internal consistency (α = 0.89 and 0.91 for PA and NA in women, respectively, and α = 0.87 and 0.89 for PA and NA in men, respectively) in college students [80].

### Secondary outcomes measures

#### Diagnosis-specific measures

Depending on each participant’s main diagnosis, different instruments will be implemented.

- OCD: Obsessive-Compulsive Inventory-Revised (OCI-R) [81]. The OCI-R is a short scale made up of 18 items rated from 1 to 4 to assess obsessive-compulsive symptoms. The OCI-R yields six subscales: washing, checking, ordering, obsessing, hoarding, and neutralizing. The OCI-R has good internal consistency (α = .81 to .93), good to excellent test-retest reliability (α = .57 to .91), good convergent validity, and a solid factor structure. The Spanish version of the OCI-R has been found to be good (α = .86) [82].
- PD/AG: Self-Reported Panic Disorder Severity Scale (PDSS-SR) [83]. The PDSS-SR is a 7-item self-report measure of panic disorder severity. This scale assesses panic attack frequency, distress during panic attacks, severity of anticipatory anxiety, fear and avoidance of agoraphobic situations, fear and avoidance of physical sensations, and work and social impairment. The scale has shown excellent reliability (α = .917), test-retest reliability (ICC = .81), and sensitivity to change. The psychometric analysis of the Spanish version showed excellent internal consistency (α = .85), good test-retest reliability, and adequate convergent validity [82].
- GAD: Penn State Worry Questionnaire (PSWQ) [84]. It is a questionnaire that evaluates symptoms related to GAD. The PSWQ is a 16-item self-report questionnaire that assesses the tendency to worry, as well as the intensity of the worry characteristic of GAD as an uncontrollable, generalized, and excessive experience. The PSWQ has demonstrated good internal consistency ranging from .91 to .95, and good validity and test-retest reliability. The Spanish version of the scale showed an internal consistency of .90 and a test-retest reliability of .82, as well as adequate convergent and discriminant validity [85].
- SAD: Social Interaction Anxiety Scale (SIAS) [86]. This scale is a 20-item self-report measure rated on a 5-point scale ranging from 0 (*not at all characteristic or true of me*) to 4 (*extremely characteristic of me*). It assesses cognitive, affective, and behavioral reactions in interactive social situations (symptoms related to social phobia). The SIAS has high internal consistency (α = .88 to .94) and good test-retest and discriminant reliability, as well as adequate construct validity. The Spanish validation of the scale showed adequate internal consistency [87].

#### Personality measures

##### NEO-five factor Inventory

The NEO FFI is the short version of the NEO-PI-R [88], designed to assess the five personality dimensions through 60 items. In this study, only the subscales of neuroticism and extraversion are used. Each scale contained 12 items with a five-point Likert response format. Two-week retest reliability is uniformly high, ranging from 0.86 to 0.90 for the five scales [89], and internal consistency ranges from 0.68 to 0.86 [89].The Spanish version of the NEO FFI has been found to be good [90].

#### Quality of life

*EuroQol 5D* (EQ-5D-Spanish version) [91] is a generic instrument for measuring health-related quality of life. It can be used in relatively healthy individuals (general population) as well as in groups of patients with different pathologies. Each individual rates his/her own health on each of the two parts of the questionnaire. In the first part, the individual must check the level corresponding to his/her state of health in each of the five domains: mobility, self-care, daily activities, pain/discomfort and anxiety/depression. Each dimension is divided into three levels of severity (without problems, some problems or moderate problems, and severe problems), yielding a population-based preference score or societal index (SI). There are 243 possible combinations - health states -, and the SI is calculated on the basis of these health states. The index value ranges from 1 (best health state) to 0 (death), although there are negative values for the index corresponding to those health states that are rated as worse than death. In the second part of the questionnaire, the individual values his/her own health on a more general visual analogical scale (VAS), a 10 cm vertical line on which the best and worst imaginable health states score 100 and 0, respectively.

#### Suicidal ideation

A suicide item has been included within the ODSIS [92] with the aim of obtaining a suicide risk indicator throughout the treatment. The total score depends on this single item, and the maximum score is 4, with a range from 0 (“*absence of thoughts of suicide*”) to 4 ("*thoughts of suicide all the time*").

#### Post-module measures

Scores on anxious and depressive symptomatology will also be obtained after each module has been completed.

##### *Overall Anxiety Severity and Impairment Scale (OASIS)* [93]

The OASIS consists of a 5-item questionnaire with a scale from 0 to 4, which measures the frequency and severity of anxiety, as well as the level of avoidance, work/academic/home interference, and social and everyday life impairment related to anxiety symptoms. The instructions tell the respondent to consider a wide range of anxiety symptoms (e.g., panic attacks, worries, flashbacks) when answering the questions, and the time frame is “during the past week”. A psychometric analysis of the OASIS scale found good internal consistency (α = .80), test-retest reliability (k = .82) and convergent validity for this instrument. The Spanish version of the OASIS confirmed the factorial structure and reliability and validity data obtained by the original authors (internal consistency in both populations, general and clinical (α = .0.86 and test-retest reliability (k = .84) [94].

##### *Overall Depression Severity and Impairment Scale (ODSIS)* [92]

The ODSIS is a self-report measure with five items. Individuals select among five different response options ranging from 0 to 4 for each item. This scale evaluates experiences related to depression. The ODSIS measures the frequency and severity of depression, as well as the level of avoidance, work/academic/home interference, and social and everyday life impairment related to depression symptoms. The ODSIS can also be used to assess severity and impairment associated with low mood. In the Spanish version of the ODSIS, the internal consistency has been shown to be excellent, with a Cronbach’s alpha between .91 and .94 and good convergent and discriminant validity [95].

#### Expectation of treatment scale and opinion of treatment scale.

The Expectation of Treatment Scale and Opinion of Treatment Scale are questionnaires adapted from Borkovec and Nau [96]. Each scale contains five items, rated from 0 (“strongly disagree”) to 10 (“strongly agree”), which cover how logical the treatment seemed, to what extent it could satisfy the patient, whether it could be used to treat other psychological problems, and its usefulness for the patient’s specific problem. The expectation scale is applied once the intervention has been explained, at the end of the “Welcome module”. This scale measures the patient’s subjective expectations about the treatment. In addition, the opinion scale is administered when the patient has completed the treatment, and its aim is to assess satisfaction with the intervention. Our group has used this questionnaire in several research studies [48, 97].

The study measures and assessment times are summarized in Table 2.

**Table 2:** Study measures and assessment times

Measure	Aim	Time of assessment
MINI Neuropsychiatric Interview	Diagnosis	BL, Post-T and FU
PANAS	Positive and negative affect	Post-T and Post-module
BDI-II	Severity of depression	BL, Post-T and FU
BAI	Severity of anxiety	BL, Post-T and FU
OCI-R	Severity of OCD symptoms	BL, Post-T and FU
PDSS-SR	Severity of PD and agoraphobia symptoms	BL, Post-T and FU
PSWQ	Severity of GAD symptoms	BL, Post-T and FU
SIAS	Severity of SAD symptoms	BL, Post-T and FU
NEO FFI	Neuroticism and Extraversion	BL, Post-T and FU
EQ-5D	Health-related quality of life	BL, Post-T and FU
OASIS	Severity of anxiety	Post-module
ODSIS	Severity of depression	Post-module
Suicide item	Suicidal ideation	BL, Post-module, Post-T and FU
Expectation of Treatment Scale	Expectation of treatment	BL
Opinion of Treatment Scale	Opinion of treatment	Post-T

*BL*, Baseline; *Post-T*, post-treatment; *FU*, follow-ups, 3 and 12-month follow-ups; *PANAS*, Positive and Negative Affect Scale; *BDI-II*, Beck Depression Inventory-II; *BAI*, Beck Anxiety Inventory; *OCI-R*, Obsessive-Compulsive Inventory-Revised; *PDSS-SR*, Self-Reported Panic Disorder Severity Scale; *PSWQ*, Penn State Worry Questionnaire; *SIAS*, Social Interaction Anxiety Scale; *NEO FFI*, NEO-Five Factor Inventory; *EQ-5D*, EuroQoL-5D questionnaire; *OASIS*, Overall Anxiety Severity and Impairment Scale; *ODSIS*, Overall Depression Severity and Impairment Scale

### Data analyses

Intention-to -treat analyses and per protocol analyses will be performed, and CONSORT recommendations will be followed [98]. First, the three groups will be compared to verify that there are no significant differences among them at baseline on the outcome measures in order to confirm that they are comparable after randomization. One-way ANOVAs for continuous variables and Chi-squared tests of independence for categorical ones will be used. For the continuous outcome measures on the posttest, the homoscedasticity assumption will be assessed with the Levene test. Where this assumption is met, the usual *F*-test will be applied to compare the posttest means for the three experimental conditions. If the homoscedasticity assumption is not met, the Brown-Forsythe *F*-test will be applied. Statistically significant *F*-tests will be followed by post hoc comparisons. In particular, the Tukey procedure will be applied where the homoscedasticity assumption is met, and the Games-Howell procedure if this assumption is not met.

The intention-to-treat principle will be used when analyzing primary and secondary post-treatment data and data collected at the 3- and 12-month follow-ups, using mixed-effect models with full information maximum likelihood estimation. This method has been recommended due to its flexibility on repeated-measures ANOVAs in handling missing data more appropriately [99]. To complement the results of the ANOVAs and post hoc comparisons, effect sizes will be calculated by using the standardized mean difference proposed by Cohen [62]. These effect sizes will be calculated to assess both within- and between-group changes, all of them based on a pooled standard deviation.

Although per protocol analyses (completers only analysis) suffer from selection bias, they will also be conducted to help to draw conclusions about the maximum treatment efficacy in patients who comply fully with the treatment [100].

When the trial ends, the analytic methodology for the RCT will be reviewed before analyzing the data in order to select the most appropriate analytic procedures.

## Discussion

This study describes a new Internet-based transdiagnostic treatment protocol for patients diagnosed with ED. One of the core aims of this study is to provide data from an RCT to evaluate the efficacy of this protocol in a community sample, compared to a waiting list group. This protocol includes the transdiagnostic cognitive-behavioral principles [17] designed for a wide range of anxiety and depressive disorders to down-regulate NA, and it also incorporates a specific treatment component to address PA. These two ways of delivering the protocol will be tested in order to explore the effect of adding a specific therapeutic component to up-regulate PA.

Moreover, the aim of the present study is to make progress in resolving some of the challenges in the field of mental health [101], specifically in understanding the necessary treatment components to modify clinical symptoms (depression and anxiety) and strengthen people’s resilience, making them less vulnerable [101, 102].The potential impact on basic personality dimensions (N/NA/BI) and (E/PA/BA) will also be studied. As Barlow suggests [102], personality dimensions may be malleable over time, and so it can be relevant to study the “malleability” of neuroticism. Likewise, the study will also examine whether it is possible to develop strategies to modify PA (extraversion). There is growing support in the literature for the claim that positive emotions promote flexible and creative thinking and play a fundamental role in the construction of psychological strengths and intellectual and social resources that can be useful in difficult situations in the future [103–106]. Some studies have proposed ways to address both the assessment and treatment of PA regulation from a transdiagnostic perspective [28, 107], but further research is needed on this topic. This study aims to explore the effect of treatment components on increasing wellbeing and PA. If this is confirmed, it would certainly represent an important shift in the research, understanding, assessment, and treatment of ED. To the best of our knowledge, this is the first study of an RCT testing an online transdiagnostic treatment protocol for ED with a specific therapeutic component to directly up-regulate PA.

The data obtained with this study can be compared to results obtained in studies with face-to-face transdiagnostic protocols using benchmarking strategies [33]. In addition, the data will also be compared to transdiagnostic protocols applied over the Internet, but without components for up-regulating PA [15, 108–112].

The objective of establishing action strategies to improve access to EBTs should also be taken into consideration, as well as the goal of providing psychological support to all those in need [5]. Undoubtedly, we are witnessing the beginning of a new era in the field of psychological treatments. They have already gone beyond traditional EBTs, and now it is possible to manage these online protocols with good results [53]. The use of technology and the Internet can help to disseminate and increase the access to these interventions.

Finally, we will test the effectiveness of the application of this Internet-based program with minimal support from the clinician, and the acceptability of this online program in patients from a community sample.

We are aware that this study has limitations. One of the most important is the different number of modules in the two protocols. We tried to control this by giving all the participants equal time and allowing them to use the program as much as they like throughout the whole process. However, in order to test the potential additional impact of the positive psychology component, it seemed necessary to compare it to the traditional transdiagnostic protocol. If the new component is found to have any effect, future research should be carried out to show that this effect is not simply due to the larger number of modules in the protocol.

There are further limitations. For example, dropout rates are expected to be high (around 30%) [53, 63]. To minimize this problem, human and ICT support will be provided. In addition, another limitation may be recruitment difficulties, to the extent that people still do not have access to the Internet at home, or they have negative attitudes towards Internet interventions.

In summary, this study aims to contribute to the literature on the efficacy of transdiagnostic approaches to ED in general, and it more specifically seeks to explore the possible impact of specific component designed to up-regulate positive affect. Despite its limitations, if positive results are achieved, they will have a clear impact on the design and application of future transdiagnostic treatment protocols for ED, as a way to more effectively address the temperament vulnerabilities, that is, the core aspects of these disorders [102].

## Abbreviations

ADNOS: Anxiety Disorder Not Otherwise Specified
AES: Advanced Encryption Standard
AG: Agoraphobia
ANOVA: Analysis of variance
BAI: Beck Anxiety Inventory
BDI-II: Beck Depression Inventory-II
BL: Baseline
CBT: Cognitive Behavior Therapy
CONSORT: Consolidated Standards Of Reporting Trials
DD: Dysthymia
DSM-IV: Diagnostic and Statistical Manual of mental disorders, fourth text
E/PA/BA: Extraversion/Positive Affect/Behavioral Activation
EBTs: Evidence-based psychological treatments
ED: Emotional disorders
EDBs: Emotion Driven Behaviors
EQ-5D: EuroQol-5D questionnaire
FU: Follow-Ups
GAD: Generalized Anxiety Disorder
ICD-10: International Classification of Diseases, tenth edition
ICT: Information and Communication Technologies
MDD: Major Depression Disorder
MDNOS: Mood Disorder Not Otherwise Specified
MINI: Mini-International Neuropsychiatric Interview
N/NA/BI: Neuroticism/Negative Affect/Behavioral Inhibition
NEO-FFI: NEO Five Factor Inventory
OASIS: Overall Anxiety and Impairment Scale
OCD: Obsessive-compulsive Disorder
OCI-R: Obsessive-Compulsive Inventory-Revised
ODSIS: Overall Depression and Impairment Scale
PANAS: Positive and Negative Affect Schedule
PD: Panic Disorder
PDSS-SR: Self-Reported Panic Disorder Severity Scale
POST-T: Post-Treatment
PSWQ: Pen State Worry Questionnaire
RCT: Randomized Controlled Trial
SAD: Social Anxiety Disorder
SI: Social Index
SIAS: Social Interaction Anxiety Inventory
TIBP: Transdiagnostic Internet-Based Protocol
TIBP + PA: Transdiagnostic Internet-Based Protocol + Positive Affect Component
UP: Unified Protocol
VAS: Visual Analogical Scale
WBT: Well-Being Therapy
WL: Waiting List
